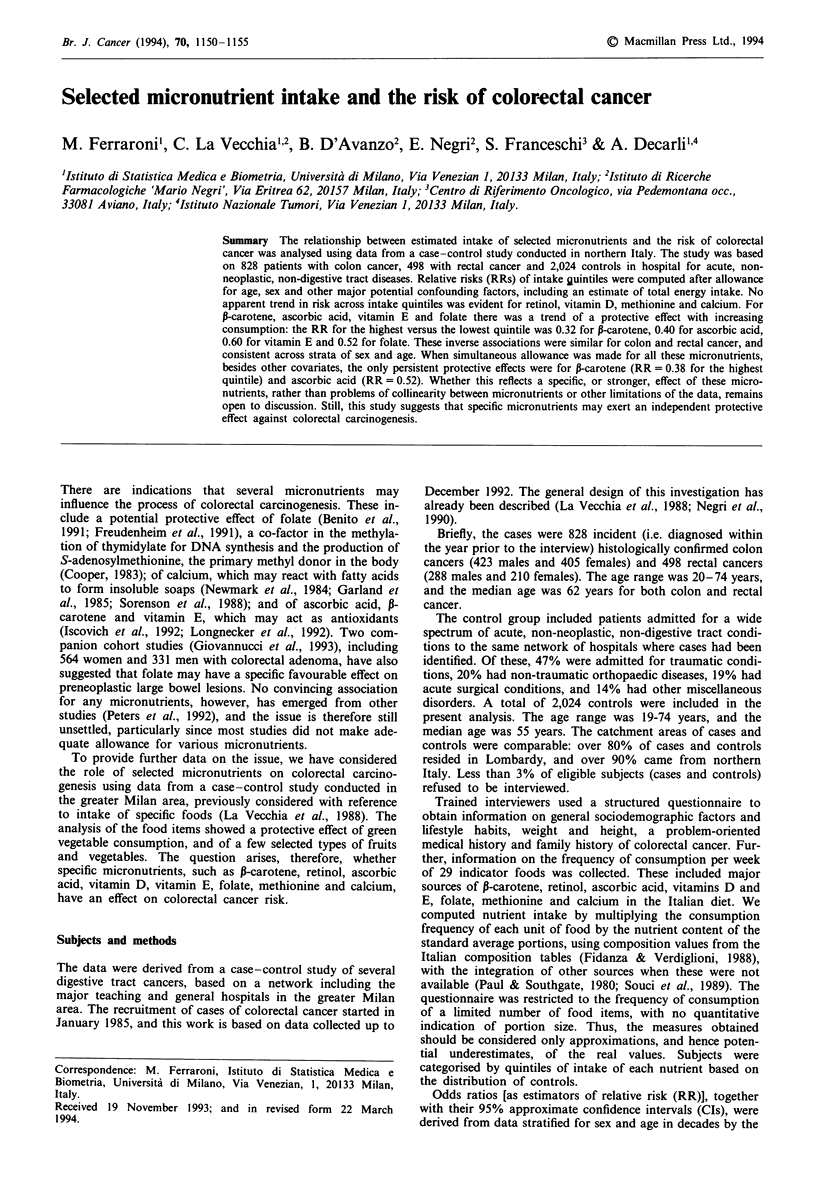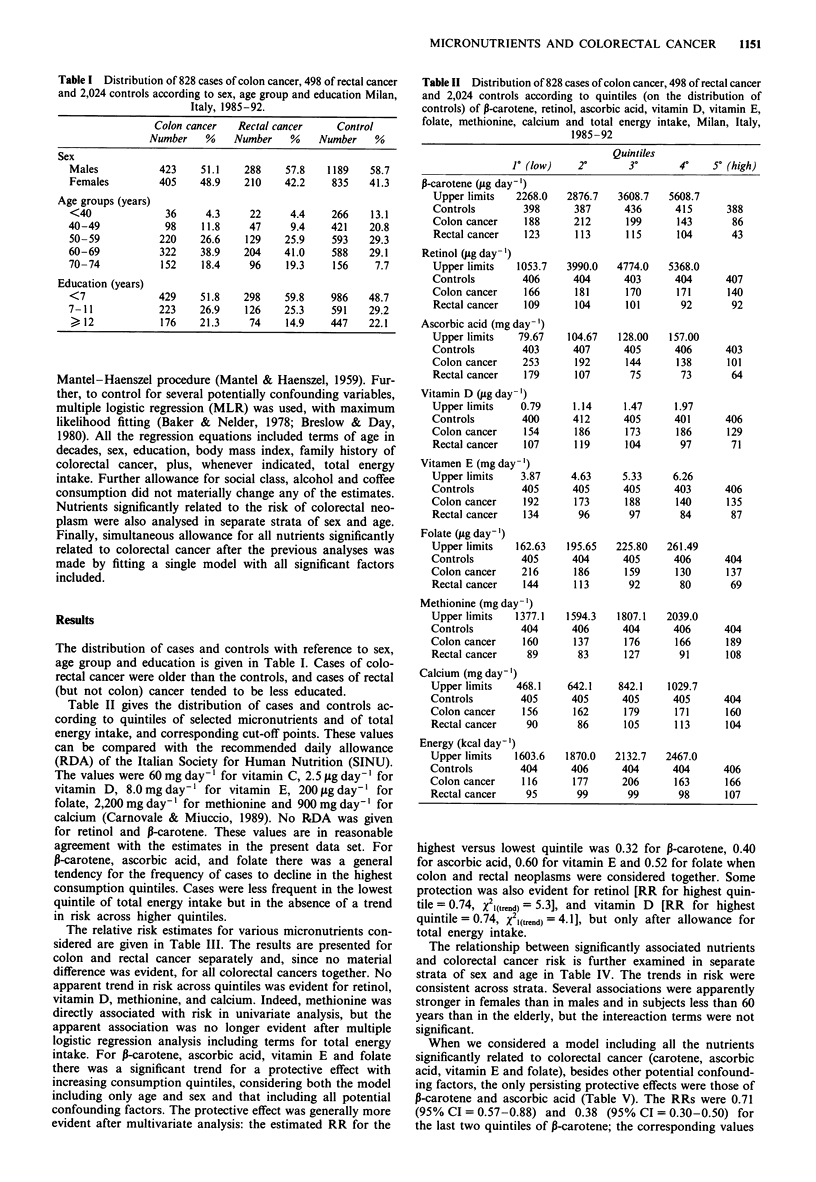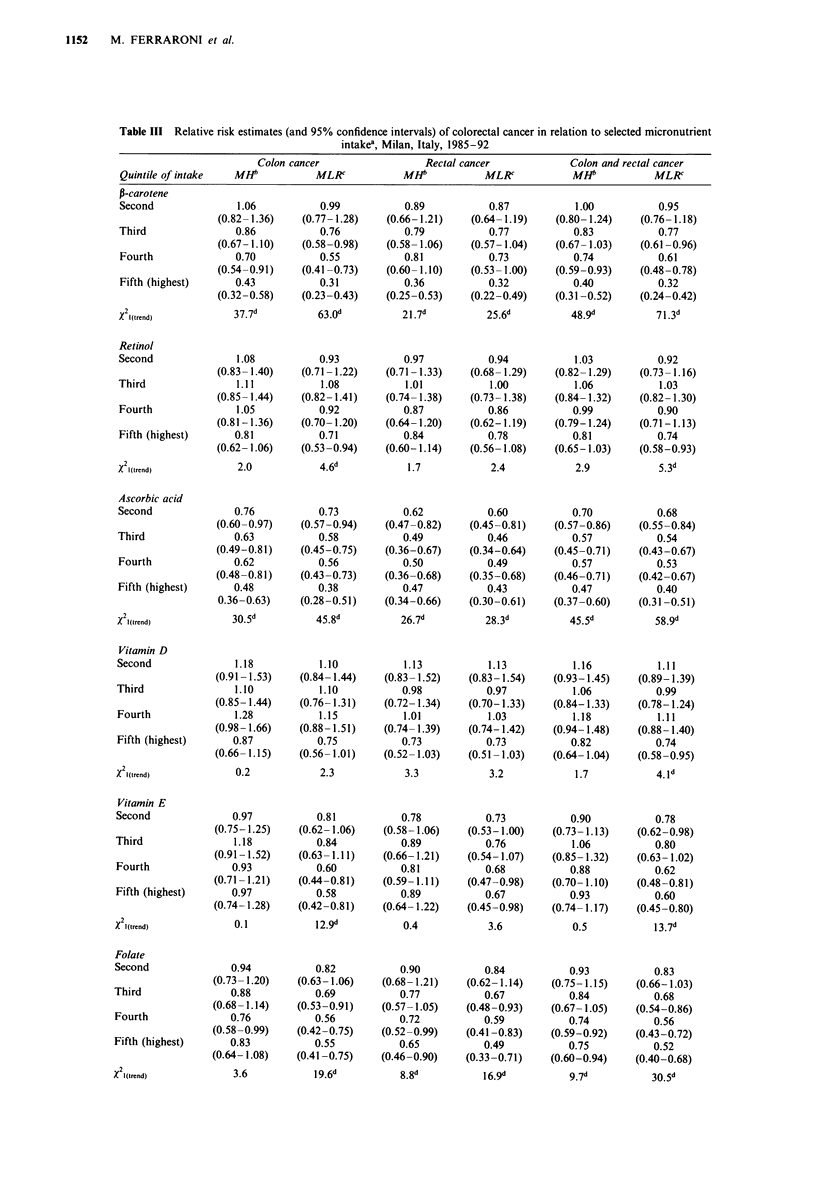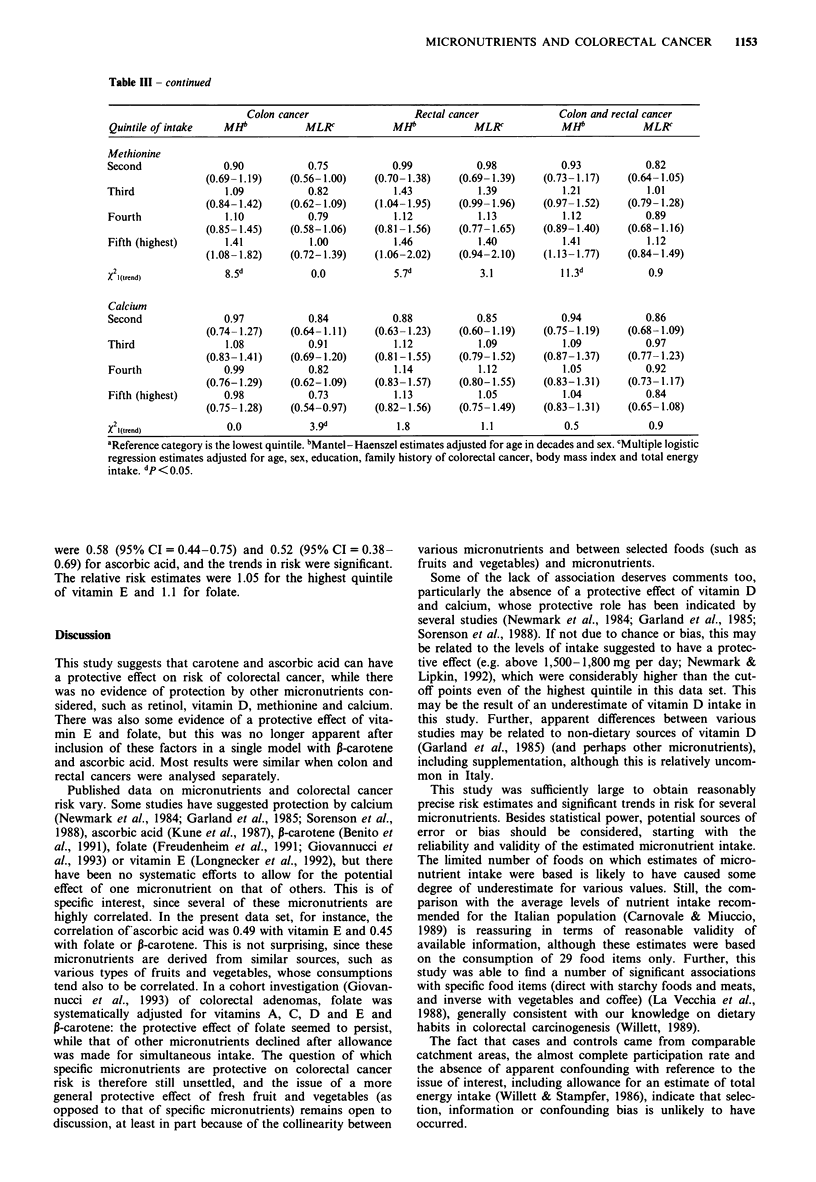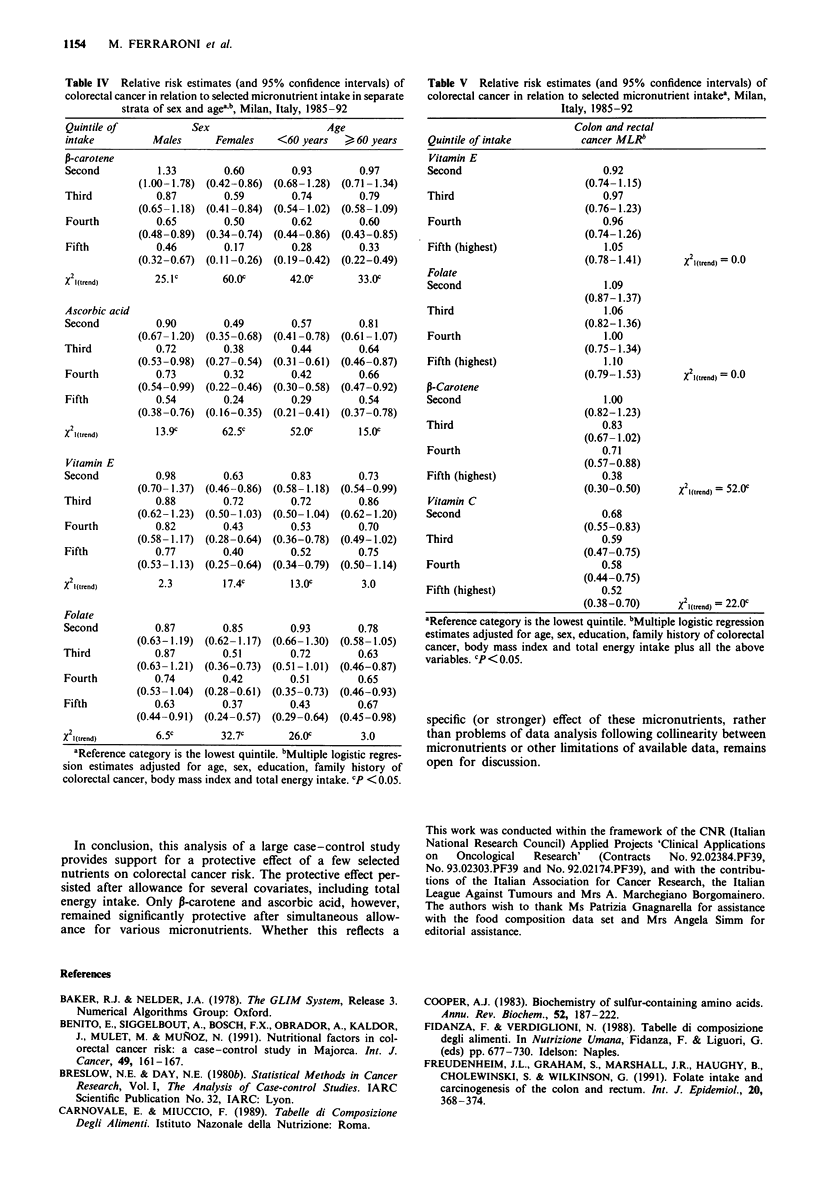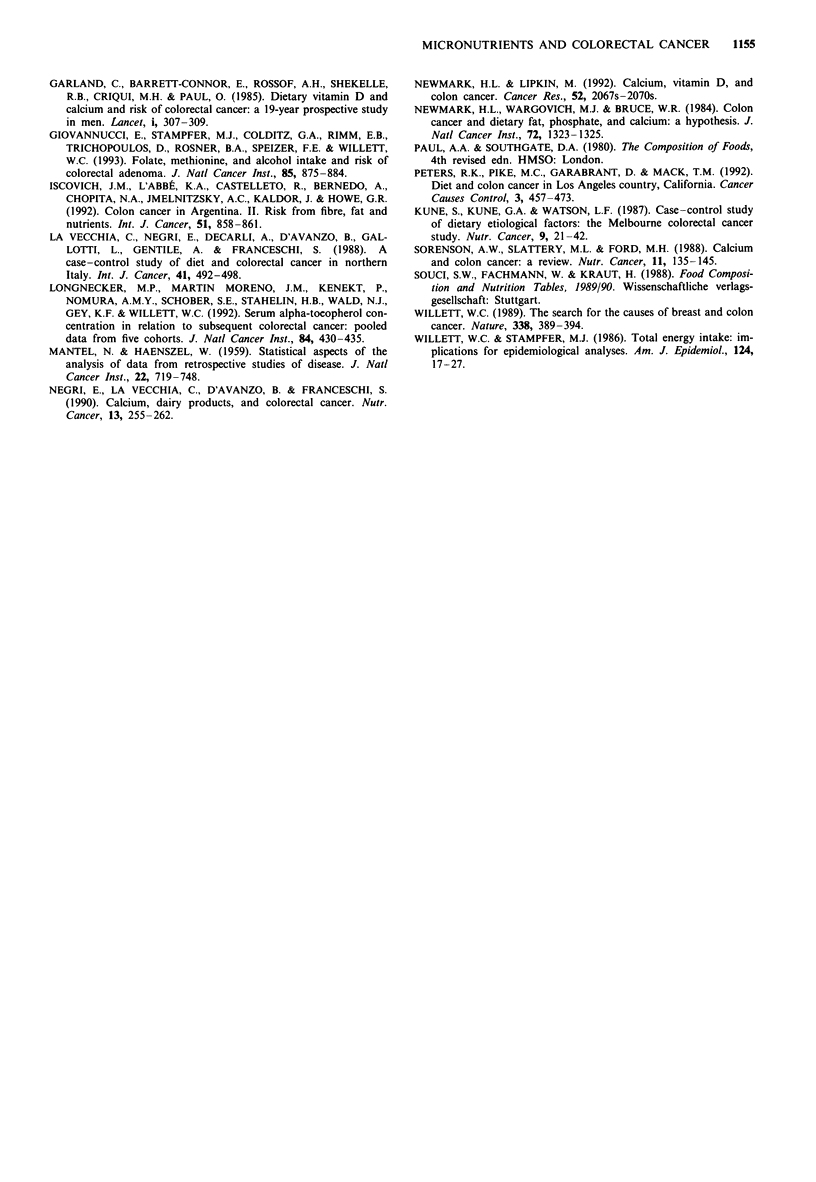# Selected micronutrient intake and the risk of colorectal cancer.

**DOI:** 10.1038/bjc.1994.463

**Published:** 1994-12

**Authors:** M. Ferraroni, C. La Vecchia, B. D'Avanzo, E. Negri, S. Franceschi, A. Decarli

**Affiliations:** Istituto di Statistica Medica e Biometria, Università di Milano, Italy.

## Abstract

The relationship between estimated intake of selected micronutrients and the risk of colorectal cancer was analysed using data from a case-control study conducted in northern Italy. The study was based on 828 patients with colon cancer, 498 with rectal cancer and 2,024 controls in hospital for acute, non-neoplastic, non-digestive tract diseases. Relative risks (RRs) of intake quintiles were computed after allowance for age, sex and other major potential confounding factors, including an estimate of total energy intake. No apparent trend in risk across intake quintiles was evident for retinol, vitamin D, methionine and calcium. For beta-carotene, ascorbic acid, vitamin E and folate there was a trend of a protective effect with increasing consumption: the RR for the highest versus the lowest quintile was 0.32 for beta-carotene, 0.40 for ascorbic acid, 0.60 for vitamin E and 0.52 for folate. These inverse associations were similar for colon and rectal cancer, and consistent across strata of sex and age. When simultaneous allowance was made for all these micronutrients, besides other covariates, the only persistent protective effects were for beta-carotene (RR = 0.38 for the highest quintile) and ascorbic acid (RR = 0.52). Whether this reflects a specific, or stronger, effect of these micronutrients, rather than problems of collinearity between micronutrients or other limitations of the data, remains open to discussion. Still, this study suggests that specific micronutrients may exert an independent protective effect against colorectal carcinogenesis.


Br. J. Cancer (1994), 70, 1150–1155 © Macmillan Press Ltd., 1994

### Selected micronutrient intake and the risk of colorectal cancer

M. Ferraroni[1], C. La Vecchia[1,2], B. D'Avanzo[2], E. Negri[2], S. Franceschi[3] & A. Decarli[1,4]

[1]*Istituto di Statistica Medica e Biometria, Università di Milano, Via Venezian 1, 20133 Milan, Italy;* [2]*Istituto di Ricerche Farmacologiche 'Mario Negri', Via Eritrea 62, 20157 Milan, Italy;* [3]*Centro di Riferimento Oncologico, via Pedemontana occ., 33081 Aviano, Italy;* [4]*Istituto Nazionale Tumori, Via Venezian 1, 20133 Milan, Italy.*

**Summary** The relationship between estimated intake of selected micronutrients and the risk of colorectal cancer was analysed using data from a case–control study conducted in northern Italy. The study was based on 828 patients with colon cancer, 498 with rectal cancer and 2,024 controls in hospital for acute, non-neoplastic, non-digestive tract diseases. Relative risks (RRs) of intake quintiles were computed after allowance for age, sex and other major potential confounding factors, including an estimate of total energy intake. No apparent trend in risk across intake quintiles was evident for retinol, vitamin D, methionine and calcium. For β-carotene, ascorbic acid, vitamin E and folate there was a trend of a protective effect with increasing consumption: the RR for the highest versus the lowest quintile was 0.32 for β-carotene, 0.40 for ascorbic acid, 0.60 for vitamin E and 0.52 for folate. These inverse associations were similar for colon and rectal cancer, and consistent across strata of sex and age. When simultaneous allowance was made for all these micronutrients, besides other covariates, the only persistent protective effects were for β-carotene (RR = 0.38 for the highest quintile) and ascorbic acid (RR = 0.52). Whether this reflects a specific, or stronger, effect of these micronutrients, rather than problems of collinearity between micronutrients or other limitations of the data, remains open to discussion. Still, this study suggests that specific micronutrients may exert an independent protective effect against colorectal carcinogenesis.

There are indications that several micronutrients may influence the process of colorectal carcinogenesis. These include a potential protective effect of folate (Benito *et al.*, 1991; Freudenheim *et al.*, 1991), a co-factor in the methylation of thymidylate for DNA synthesis and the production of *S*-adenosylmethionine, the primary methyl donor in the body (Cooper, 1983); of calcium, which may react with fatty acids to form insoluble soaps (Newmark *et al.*, 1984; Garland *et al.*, 1985; Sorenson *et al.*, 1988); and of ascorbic acid, β-carotene and vitamin E, which may act as antioxidants (Iscovich *et al.*, 1992; Longnecker *et al.*, 1992). Two companion cohort studies (Giovannucci *et al.*, 1993), including 564 women and 331 men with colorectal adenoma, have also suggested that folate may have a specific favourable effect on preneoplastic large bowel lesions. No convincing association for any micronutrients, however, has emerged from other studies (Peters *et al.*, 1992), and the issue is therefore still unsettled, particularly since most studies did not make adequate allowance for various micronutrients.

To provide further data on the issue, we have considered the role of selected micronutrients on colorectal carcinogenesis using data from a case–control study conducted in the greater Milan area, previously considered with reference to intake of specific foods (La Vecchia *et al.*, 1988). The analysis of the food items showed a protective effect of green vegetable consumption, and of a few selected types of fruits and vegetables. The question arises, therefore, whether specific micronutrients, such as β-carotene, retinol, ascorbic acid, vitamin D, vitamin E, folate, methionine and calcium, have an effect on colorectal cancer risk.

### Subjects and methods

The data were derived from a case–control study of several digestive tract cancers, based on a network including the major teaching and general hospitals in the greater Milan area. The recruitment of cases of colorectal cancer started in January 1985, and this work is based on data collected up to December 1992. The general design of this investigation has already been described (La Vecchia *et al.*, 1988; Negri *et al.*, 1990).

Briefly, the cases were 828 incident (i.e. diagnosed within the year prior to the interview) histologically confirmed colon cancers (423 males and 405 females) and 498 rectal cancers (288 males and 210 females). The age range was 20–74 years, and the median age was 62 years for both colon and rectal cancer.

The control group included patients admitted for a wide spectrum of acute, non-neoplastic, non-digestive tract conditions to the same network of hospitals where cases had been identified. Of these, 47% were admitted for traumatic conditions, 20% had non-traumatic orthopaedic diseases, 19% had acute surgical conditions, and 14% had other miscellaneous disorders. A total of 2,024 controls were included in the present analysis. The age range was 19-74 years, and the median age was 55 years. The catchment areas of cases and controls were comparable: over 80% of cases and controls resided in Lombardy, and over 90% came from northern Italy. Less than 3% of eligible subjects (cases and controls) refused to be interviewed.

Trained interviewers used a structured questionnaire to obtain information on general sociodemographic factors and lifestyle habits, weight and height, a problem-oriented medical history and family history of colorectal cancer. Further, information on the frequency of consumption per week of 29 indicator foods was collected. These included major sources of β-carotene, retinol, ascorbic acid, vitamins D and E, folate, methionine and calcium in the Italian diet. We computed nutrient intake by multiplying the consumption frequency of each unit of food by the nutrient content of the standard average portions, using composition values from the Italian composition tables (Fidanza & Verdiglioni, 1988), with the integration of other sources when these were not available (Paul & Southgate, 1980; Souci *et al.*, 1989). The questionnaire was restricted to the frequency of consumption of a limited number of food items, with no quantitative indication of portion size. Thus, the measures obtained should be considered only approximations, and hence potential underestimates, of the real values. Subjects were categorised by quintiles of intake of each nutrient based on the distribution of controls.

Odds ratios [as estimators of relative risk (RR)], together with their 95% approximate confidence intervals (CIs), were derived from data stratified for sex and age in decades by the

Correspondence: M. Ferraroni, Istituto di Statistica Medica e Biometria, Università di Milano, Via Venezian, 1, 20133 Milan, Italy.
Received 19 November 1993; and in revised form 22 March 1994.

**Table I** Distribution of 828 cases of colon cancer, 498 of rectal cancer and 2,024 controls according to sex, age group and education Milan, Italy, 1985-92.

| | *Colon cancer* Number | % | *Rectal cancer* Number | % | *Control* Number | % |
|---|---|---|---|---|---|---|
| Sex | | | | | | |
| Males | 423 | 51.1 | 288 | 57.8 | 1189 | 58.7 |
| Females | 405 | 48.9 | 210 | 42.2 | 835 | 41.3 |
| Age groups (years) | | | | | | |
| <40 | 36 | 4.3 | 22 | 4.4 | 266 | 13.1 |
| 40-49 | 98 | 11.8 | 47 | 9.4 | 421 | 20.8 |
| 50-59 | 220 | 26.6 | 129 | 25.9 | 593 | 29.3 |
| 60-69 | 322 | 38.9 | 204 | 41.0 | 588 | 29.1 |
| 70-74 | 152 | 18.4 | 96 | 19.3 | 156 | 7.7 |
| Education (years) | | | | | | |
| <7 | 429 | 51.8 | 298 | 59.8 | 986 | 48.7 |
| 7-11 | 223 | 26.9 | 126 | 25.3 | 591 | 29.2 |
| ⩾12 | 176 | 21.3 | 74 | 14.9 | 447 | 22.1 |

Mantel-Haenszel procedure (Mantel & Haenszel, 1959). Further, to control for several potentially confounding variables, multiple logistic regression (MLR) was used, with maximum likelihood fitting (Baker & Nelder, 1978; Breslow & Day, 1980). All the regression equations included terms of age in decades, sex, education, body mass index, family history of colorectal cancer, plus, whenever indicated, total energy intake. Further allowance for social class, alcohol and coffee consumption did not materially change any of the estimates. Nutrients significantly related to the risk of colorectal neoplasm were also analysed in separate strata of sex and age. Finally, simultaneous allowance for all nutrients significantly related to colorectal cancer after the previous analyses was made by fitting a single model with all significant factors included.

### Results

The distribution of cases and controls with reference to sex, age group and education is given in Table I. Cases of colorectal cancer were older than the controls, and cases of rectal (but not colon) cancer tended to be less educated.

Table II gives the distribution of cases and controls according to quintiles of selected micronutrients and of total energy intake, and corresponding cut-off points. These values can be compared with the recommended daily allowance (RDA) of the Italian Society for Human Nutrition (SINU). The values were 60 mg day$^{-1}$ for vitamin C, 2.5 μg day$^{-1}$ for vitamin D, 8.0 mg day$^{-1}$ for vitamin E, 200 μg day$^{-1}$ for folate, 2,200 mg day$^{-1}$ for methionine and 900 mg day$^{-1}$ for calcium (Carnovale & Miuccio, 1989). No RDA was given for retinol and β-carotene. These values are in reasonable agreement with the estimates in the present data set. For β-carotene, ascorbic acid, and folate there was a general tendency for the frequency of cases to decline in the highest consumption quintiles. Cases were less frequent in the lowest quintile of total energy intake but in the absence of a trend in risk across higher quintiles.

The relative risk estimates for various micronutrients considered are given in Table III. The results are presented for colon and rectal cancer separately and, since no material difference was evident, for all colorectal cancers together. No apparent trend in risk across quintiles was evident for retinol, vitamin D, methionine, and calcium. Indeed, methionine was directly associated with risk in univariate analysis, but the apparent association was no longer evident after multiple logistic regression analysis including terms for total energy intake. For β-carotene, ascorbic acid, vitamin E and folate there was a significant trend for a protective effect with increasing consumption quintiles, considering both the model including only age and sex and that including all potential confounding factors. The protective effect was generally more evident after multivariate analysis: the estimated RR for the

**Table II** Distribution of 828 cases of colon cancer, 498 of rectal cancer and 2,024 controls according to quintiles (on the distribution of controls) of β-carotene, retinol, ascorbic acid, vitamin D, vitamin E, folate, methionine, calcium and total energy intake, Milan, Italy, 1985-92

| | *Quintiles* *1° (low)* | *2°* | *3°* | *4°* | *5° (high)* |
|---|---|---|---|---|---|
| β-carotene (μg day$^{-1}$) | | | | | |
| Upper limits | 2268.0 | 2876.7 | 3608.7 | 5608.7 | |
| Controls | 398 | 387 | 436 | 415 | 388 |
| Colon cancer | 188 | 212 | 199 | 143 | 86 |
| Rectal cancer | 123 | 113 | 115 | 104 | 43 |
| Retinol (μg day$^{-1}$) | | | | | |
| Upper limits | 1053.7 | 3990.0 | 4774.0 | 5368.0 | |
| Controls | 406 | 404 | 403 | 404 | 407 |
| Colon cancer | 166 | 181 | 170 | 171 | 140 |
| Rectal cancer | 109 | 104 | 101 | 92 | 92 |
| Ascorbic acid (mg day$^{-1}$) | | | | | |
| Upper limits | 79.67 | 104.67 | 128.00 | 157.00 | |
| Controls | 403 | 407 | 405 | 406 | 403 |
| Colon cancer | 253 | 192 | 144 | 138 | 101 |
| Rectal cancer | 179 | 107 | 75 | 73 | 64 |
| Vitamin D (μg day$^{-1}$) | | | | | |
| Upper limits | 0.79 | 1.14 | 1.47 | 1.97 | |
| Controls | 400 | 412 | 405 | 401 | 406 |
| Colon cancer | 154 | 186 | 173 | 186 | 129 |
| Rectal cancer | 107 | 119 | 104 | 97 | 71 |
| Vitamen E (mg day$^{-1}$) | | | | | |
| Upper limits | 3.87 | 4.63 | 5.33 | 6.26 | |
| Controls | 405 | 405 | 405 | 403 | 406 |
| Colon cancer | 192 | 173 | 188 | 140 | 135 |
| Rectal cancer | 134 | 96 | 97 | 84 | 87 |
| Folate (μg day$^{-1}$) | | | | | |
| Upper limits | 162.63 | 195.65 | 225.80 | 261.49 | |
| Controls | 405 | 404 | 405 | 406 | 404 |
| Colon cancer | 216 | 186 | 159 | 130 | 137 |
| Rectal cancer | 144 | 113 | 92 | 80 | 69 |
| Methionine (mg day$^{-1}$) | | | | | |
| Upper limits | 1377.1 | 1594.3 | 1807.1 | 2039.0 | |
| Controls | 404 | 406 | 404 | 406 | 404 |
| Colon cancer | 160 | 137 | 176 | 166 | 189 |
| Rectal cancer | 89 | 83 | 127 | 91 | 108 |
| Calcium (mg day$^{-1}$) | | | | | |
| Upper limits | 468.1 | 642.1 | 842.1 | 1029.7 | |
| Controls | 405 | 405 | 405 | 405 | 404 |
| Colon cancer | 156 | 162 | 179 | 171 | 160 |
| Rectal cancer | 90 | 86 | 105 | 113 | 104 |
| Energy (kcal day$^{-1}$) | | | | | |
| Upper limits | 1603.6 | 1870.0 | 2132.7 | 2467.0 | |
| Controls | 404 | 406 | 404 | 404 | 406 |
| Colon cancer | 116 | 177 | 206 | 163 | 166 |
| Rectal cancer | 95 | 99 | 99 | 98 | 107 |

highest versus lowest quintile was 0.32 for β-carotene, 0.40 for ascorbic acid, 0.60 for vitamin E and 0.52 for folate when colon and rectal neoplasms were considered together. Some protection was also evident for retinol [RR for highest quintile = 0.74, $\chi^2_{1(\text{trend})} = 5.3$], and vitamin D [RR for highest quintile = 0.74, $\chi^2_{1(\text{trend})} = 4.1$], but only after allowance for total energy intake.

The relationship between significantly associated nutrients and colorectal cancer risk is further examined in separate strata of sex and age in Table IV. The trends in risk were consistent across strata. Several associations were apparently stronger in females than in males and in subjects less than 60 years than in the elderly, but the intereaction terms were not significant.

When we considered a model including all the nutrients significantly related to colorectal cancer (carotene, ascorbic acid, vitamin E and folate), besides other potential confounding factors, the only persisting protective effects were those of β-carotene and ascorbic acid (Table V). The RRs were 0.71 (95% CI = 0.57-0.88) and 0.38 (95% CI = 0.30-0.50) for the last two quintiles of β-carotene; the corresponding values

**Table III** Relative risk estimates (and 95% confidence intervals) of colorectal cancer in relation to selected micronutrient intake[a], Milan, Italy, 1985–92

| | Colon cancer | | Rectal cancer | | Colon and rectal cancer | |
|---|---|---|---|---|---|---|
| *Quintile of intake* | *MH*[b] | *MLR*[c] | *MH*[b] | *MLR*[c] | *MH*[b] | *MLR*[c] |
| *β-carotene* | | | | | | |
| Second | 1.06 (0.82–1.36) | 0.99 (0.77–1.28) | 0.89 (0.66–1.21) | 0.87 (0.64–1.19) | 1.00 (0.80–1.24) | 0.95 (0.76–1.18) |
| Third | 0.86 (0.67–1.10) | 0.76 (0.58–0.98) | 0.79 (0.58–1.06) | 0.77 (0.57–1.04) | 0.83 (0.67–1.03) | 0.77 (0.61–0.96) |
| Fourth | 0.70 (0.54–0.91) | 0.55 (0.41–0.73) | 0.81 (0.60–1.10) | 0.73 (0.53–1.00) | 0.74 (0.59–0.93) | 0.61 (0.48–0.78) |
| Fifth (highest) | 0.43 (0.32–0.58) | 0.31 (0.23–0.43) | 0.36 (0.25–0.53) | 0.32 (0.22–0.49) | 0.40 (0.31–0.52) | 0.32 (0.24–0.42) |
| $\chi^2_{1(\text{trend})}$ | 37.7[d] | 63.0[d] | 21.7[d] | 25.6[d] | 48.9[d] | 71.3[d] |
| *Retinol* | | | | | | |
| Second | 1.08 (0.83–1.40) | 0.93 (0.71–1.22) | 0.97 (0.71–1.33) | 0.94 (0.68–1.29) | 1.03 (0.82–1.29) | 0.92 (0.73–1.16) |
| Third | 1.11 (0.85–1.44) | 1.08 (0.82–1.41) | 1.01 (0.74–1.38) | 1.00 (0.73–1.38) | 1.06 (0.84–1.32) | 1.03 (0.82–1.30) |
| Fourth | 1.05 (0.81–1.36) | 0.92 (0.70–1.20) | 0.87 (0.64–1.20) | 0.86 (0.62–1.19) | 0.99 (0.79–1.24) | 0.90 (0.71–1.13) |
| Fifth (highest) | 0.81 (0.62–1.06) | 0.71 (0.53–0.94) | 0.84 (0.60–1.14) | 0.78 (0.56–1.08) | 0.81 (0.65–1.03) | 0.74 (0.58–0.93) |
| $\chi^2_{1(\text{trend})}$ | 2.0 | 4.6[d] | 1.7 | 2.4 | 2.9 | 5.3[d] |
| *Ascorbic acid* | | | | | | |
| Second | 0.76 (0.60–0.97) | 0.73 (0.57–0.94) | 0.62 (0.47–0.82) | 0.60 (0.45–0.81) | 0.70 (0.57–0.86) | 0.68 (0.55–0.84) |
| Third | 0.63 (0.49–0.81) | 0.58 (0.45–0.75) | 0.49 (0.36–0.67) | 0.46 (0.34–0.64) | 0.57 (0.45–0.71) | 0.54 (0.43–0.67) |
| Fourth | 0.62 (0.48–0.81) | 0.56 (0.43–0.73) | 0.50 (0.36–0.68) | 0.49 (0.35–0.68) | 0.57 (0.46–0.71) | 0.53 (0.42–0.67) |
| Fifth (highest) | 0.48 0.36–0.63) | 0.38 (0.28–0.51) | 0.47 (0.34–0.66) | 0.43 (0.30–0.61) | 0.47 (0.37–0.60) | 0.40 (0.31–0.51) |
| $\chi^2_{1(\text{trend})}$ | 30.5[d] | 45.8[d] | 26.7[d] | 28.3[d] | 45.5[d] | 58.9[d] |
| *Vitamin D* | | | | | | |
| Second | 1.18 (0.91–1.53) | 1.10 (0.84–1.44) | 1.13 (0.83–1.52) | 1.13 (0.83–1.54) | 1.16 (0.93–1.45) | 1.11 (0.89–1.39) |
| Third | 1.10 (0.85–1.44) | 1.10 (0.76–1.31) | 0.98 (0.72–1.34) | 0.97 (0.70–1.33) | 1.06 (0.84–1.33) | 0.99 (0.78–1.24) |
| Fourth | 1.28 (0.98–1.66) | 1.15 (0.88–1.51) | 1.01 (0.74–1.39) | 1.03 (0.74–1.42) | 1.18 (0.94–1.48) | 1.11 (0.88–1.40) |
| Fifth (highest) | 0.87 (0.66–1.15) | 0.75 (0.56–1.01) | 0.73 (0.52–1.03) | 0.73 (0.51–1.03) | 0.82 (0.64–1.04) | 0.74 (0.58–0.95) |
| $\chi^2_{1(\text{trend})}$ | 0.2 | 2.3 | 3.3 | 3.2 | 1.7 | 4.1[d] |
| *Vitamin E* | | | | | | |
| Second | 0.97 (0.75–1.25) | 0.81 (0.62–1.06) | 0.78 (0.58–1.06) | 0.73 (0.53–1.00) | 0.90 (0.73–1.13) | 0.78 (0.62–0.98) |
| Third | 1.18 (0.91–1.52) | 0.84 (0.63–1.11) | 0.89 (0.66–1.21) | 0.76 (0.54–1.07) | 1.06 (0.85–1.32) | 0.80 (0.63–1.02) |
| Fourth | 0.93 (0.71–1.21) | 0.60 (0.44–0.81) | 0.81 (0.59–1.11) | 0.68 (0.47–0.98) | 0.88 (0.70–1.10) | 0.62 (0.48–0.81) |
| Fifth (highest) | 0.97 (0.74–1.28) | 0.58 (0.42–0.81) | 0.89 (0.64–1.22) | 0.67 (0.45–0.98) | 0.93 (0.74–1.17) | 0.60 (0.45–0.80) |
| $\chi^2_{1(\text{trend})}$ | 0.1 | 12.9[d] | 0.4 | 3.6 | 0.5 | 13.7[d] |
| *Folate* | | | | | | |
| Second | 0.94 (0.73–1.20) | 0.82 (0.63–1.06) | 0.90 (0.68–1.21) | 0.84 (0.62–1.14) | 0.93 (0.75–1.15) | 0.83 (0.66–1.03) |
| Third | 0.88 (0.68–1.14) | 0.69 (0.53–0.91) | 0.77 (0.57–1.05) | 0.67 (0.48–0.93) | 0.84 (0.67–1.05) | 0.68 (0.54–0.86) |
| Fourth | 0.76 (0.58–0.99) | 0.56 (0.42–0.75) | 0.72 (0.52–0.99) | 0.59 (0.41–0.83) | 0.74 (0.59–0.92) | 0.56 (0.43–0.72) |
| Fifth (highest) | 0.83 (0.64–1.08) | 0.55 (0.41–0.75) | 0.65 (0.46–0.90) | 0.49 (0.33–0.71) | 0.75 (0.60–0.94) | 0.52 (0.40–0.68) |
| $\chi^2_{1(\text{trend})}$ | 3.6 | 19.6[d] | 8.8[d] | 16.9[d] | 9.7[d] | 30.5[d] |

**Table III** – *continued*

| Quintile of intake | Colon cancer MH[b] | Colon cancer MLR[c] | Rectal cancer MH[b] | Rectal cancer MLR[c] | Colon and rectal cancer MH[b] | Colon and rectal cancer MLR[c] |
|---|---|---|---|---|---|---|
| *Methionine* | | | | | | |
| Second | 0.90 (0.69–1.19) | 0.75 (0.56–1.00) | 0.99 (0.70–1.38) | 0.98 (0.69–1.39) | 0.93 (0.73–1.17) | 0.82 (0.64–1.05) |
| Third | 1.09 (0.84–1.42) | 0.82 (0.62–1.09) | 1.43 (1.04–1.95) | 1.39 (0.99–1.96) | 1.21 (0.97–1.52) | 1.01 (0.79–1.28) |
| Fourth | 1.10 (0.85–1.45) | 0.79 (0.58–1.06) | 1.12 (0.81–1.56) | 1.13 (0.77–1.65) | 1.12 (0.89–1.40) | 0.89 (0.68–1.16) |
| Fifth (highest) | 1.41 (1.08–1.82) | 1.00 (0.72–1.39) | 1.46 (1.06–2.02) | 1.40 (0.94–2.10) | 1.41 (1.13–1.77) | 1.12 (0.84–1.49) |
| $\chi^2_{1(trend)}$ | 8.5[d] | 0.0 | 5.7[d] | 3.1 | 11.3[d] | 0.9 |
| *Calcium* | | | | | | |
| Second | 0.97 (0.74–1.27) | 0.84 (0.64–1.11) | 0.88 (0.63–1.23) | 0.85 (0.60–1.19) | 0.94 (0.75–1.19) | 0.86 (0.68–1.09) |
| Third | 1.08 (0.83–1.41) | 0.91 (0.69–1.20) | 1.12 (0.81–1.55) | 1.09 (0.79–1.52) | 1.09 (0.87–1.37) | 0.97 (0.77–1.23) |
| Fourth | 0.99 (0.76–1.29) | 0.82 (0.62–1.09) | 1.14 (0.83–1.57) | 1.12 (0.80–1.55) | 1.05 (0.83–1.31) | 0.92 (0.73–1.17) |
| Fifth (highest) | 0.98 (0.75–1.28) | 0.73 (0.54–0.97) | 1.13 (0.82–1.56) | 1.05 (0.75–1.49) | 1.04 (0.83–1.31) | 0.84 (0.65–1.08) |
| $\chi^2_{1(trend)}$ | 0.0 | 3.9[d] | 1.8 | 1.1 | 0.5 | 0.9 |

[a]Reference category is the lowest quintile. [b]Mantel–Haenszel estimates adjusted for age in decades and sex. [c]Multiple logistic regression estimates adjusted for age, sex, education, family history of colorectal cancer, body mass index and total energy intake. [d]$P < 0.05$.

were 0.58 (95% CI = 0.44–0.75) and 0.52 (95% CI = 0.38–0.69) for ascorbic acid, and the trends in risk were significant. The relative risk estimates were 1.05 for the highest quintile of vitamin E and 1.1 for folate.

### Discussion

This study suggests that carotene and ascorbic acid can have a protective effect on risk of colorectal cancer, while there was no evidence of protection by other micronutrients considered, such as retinol, vitamin D, methionine and calcium. There was also some evidence of a protective effect of vitamin E and folate, but this was no longer apparent after inclusion of these factors in a single model with β-carotene and ascorbic acid. Most results were similar when colon and rectal cancers were analysed separately.

Published data on micronutrients and colorectal cancer risk vary. Some studies have suggested protection by calcium (Newmark *et al.*, 1984; Garland *et al.*, 1985; Sorenson *et al.*, 1988), ascorbic acid (Kune *et al.*, 1987), β-carotene (Benito *et al.*, 1991), folate (Freudenheim *et al.*, 1991; Giovannucci *et al.*, 1993) or vitamin E (Longnecker *et al.*, 1992), but there have been no systematic efforts to allow for the potential effect of one micronutrient on that of others. This is of specific interest, since several of these micronutrients are highly correlated. In the present data set, for instance, the correlation of ascorbic acid was 0.49 with vitamin E and 0.45 with folate or β-carotene. This is not surprising, since these micronutrients are derived from similar sources, such as various types of fruits and vegetables, whose consumptions tend also to be correlated. In a cohort investigation (Giovannucci *et al.*, 1993) of colorectal adenomas, folate was systematically adjusted for vitamins A, C, D and E and β-carotene: the protective effect of folate seemed to persist, while that of other micronutrients declined after allowance was made for simultaneous intake. The question of which specific micronutrients are protective on colorectal cancer risk is therefore still unsettled, and the issue of a more general protective effect of fresh fruit and vegetables (as opposed to that of specific micronutrients) remains open to discussion, at least in part because of the collinearity between various micronutrients and between selected foods (such as fruits and vegetables) and micronutrients.

Some of the lack of association deserves comments too, particularly the absence of a protective effect of vitamin D and calcium, whose protective role has been indicated by several studies (Newmark *et al.*, 1984; Garland *et al.*, 1985; Sorenson *et al.*, 1988). If not due to chance or bias, this may be related to the levels of intake suggested to have a protective effect (e.g. above 1,500–1,800 mg per day; Newmark & Lipkin, 1992), which were considerably higher than the cut-off points even of the highest quintile in this data set. This may be the result of an underestimate of vitamin D intake in this study. Further, apparent differences between various studies may be related to non-dietary sources of vitamin D (Garland *et al.*, 1985) (and perhaps other micronutrients), including supplementation, although this is relatively uncommon in Italy.

This study was sufficiently large to obtain reasonably precise risk estimates and significant trends in risk for several micronutrients. Besides statistical power, potential sources of error or bias should be considered, starting with the reliability and validity of the estimated micronutrient intake. The limited number of foods on which estimates of micronutrient intake were based is likely to have caused some degree of underestimate for various values. Still, the comparison with the average levels of nutrient intake recommended for the Italian population (Carnovale & Miuccio, 1989) is reassuring in terms of reasonable validity of available information, although these estimates were based on the consumption of 29 food items only. Further, this study was able to find a number of significant associations with specific food items (direct with starchy foods and meats, and inverse with vegetables and coffee) (La Vecchia *et al.*, 1988), generally consistent with our knowledge on dietary habits in colorectal carcinogenesis (Willett, 1989).

The fact that cases and controls came from comparable catchment areas, the almost complete participation rate and the absence of apparent confounding with reference to the issue of interest, including allowance for an estimate of total energy intake (Willett & Stampfer, 1986), indicate that selection, information or confounding bias is unlikely to have occurred.

**Table IV** Relative risk estimates (and 95% confidence intervals) of colorectal cancer in relation to selected micronutrient intake in separate strata of sex and age[a,b], Milan, Italy, 1985–92

| *Quintile of intake* | *Sex* Males | Females | *Age* <60 years | ≥60 years |
|---|---|---|---|---|
| *β-carotene* | | | | |
| Second | 1.33 (1.00–1.78) | 0.60 (0.42–0.86) | 0.93 (0.68–1.28) | 0.97 (0.71–1.34) |
| Third | 0.87 (0.65–1.18) | 0.59 (0.41–0.84) | 0.74 (0.54–1.02) | 0.79 (0.58–1.09) |
| Fourth | 0.65 (0.48–0.89) | 0.50 (0.34–0.74) | 0.62 (0.44–0.86) | 0.60 (0.43–0.85) |
| Fifth | 0.46 (0.32–0.67) | 0.17 (0.11–0.26) | 0.28 (0.19–0.42) | 0.33 (0.22–0.49) |
| $\chi^2_{1(trend)}$ | 25.1[c] | 60.0[c] | 42.0[c] | 33.0[c] |
| *Ascorbic acid* | | | | |
| Second | 0.90 (0.67–1.20) | 0.49 (0.35–0.68) | 0.57 (0.41–0.78) | 0.81 (0.61–1.07) |
| Third | 0.72 (0.53–0.98) | 0.38 (0.27–0.54) | 0.44 (0.31–0.61) | 0.64 (0.46–0.87) |
| Fourth | 0.73 (0.54–0.99) | 0.32 (0.22–0.46) | 0.42 (0.30–0.58) | 0.66 (0.47–0.92) |
| Fifth | 0.54 (0.38–0.76) | 0.24 (0.16–0.35) | 0.29 (0.21–0.41) | 0.54 (0.37–0.78) |
| $\chi^2_{1(trend)}$ | 13.9[c] | 62.5[c] | 52.0[c] | 15.0[c] |
| *Vitamin E* | | | | |
| Second | 0.98 (0.70–1.37) | 0.63 (0.46–0.86) | 0.83 (0.58–1.18) | 0.73 (0.54–0.99) |
| Third | 0.88 (0.62–1.23) | 0.72 (0.50–1.03) | 0.72 (0.50–1.04) | 0.86 (0.62–1.20) |
| Fourth | 0.82 (0.58–1.17) | 0.43 (0.28–0.64) | 0.53 (0.36–0.78) | 0.70 (0.49–1.02) |
| Fifth | 0.77 (0.53–1.13) | 0.40 (0.25–0.64) | 0.52 (0.34–0.79) | 0.75 (0.50–1.14) |
| $\chi^2_{1(trend)}$ | 2.3 | 17.4[c] | 13.0[c] | 3.0 |
| *Folate* | | | | |
| Second | 0.87 (0.63–1.19) | 0.85 (0.62–1.17) | 0.93 (0.66–1.30) | 0.78 (0.58–1.05) |
| Third | 0.87 (0.63–1.21) | 0.51 (0.36–0.73) | 0.72 (0.51–1.01) | 0.63 (0.46–0.87) |
| Fourth | 0.74 (0.53–1.04) | 0.42 (0.28–0.61) | 0.51 (0.35–0.73) | 0.65 (0.46–0.93) |
| Fifth | 0.63 (0.44–0.91) | 0.37 (0.24–0.57) | 0.43 (0.29–0.64) | 0.67 (0.45–0.98) |
| $\chi^2_{1(trend)}$ | 6.5[c] | 32.7[c] | 26.0[c] | 3.0 |

[a]Reference category is the lowest quintile. [b]Multiple logistic regression estimates adjusted for age, sex, education, family history of colorectal cancer, body mass index and total energy intake. [c]$P < 0.05$.

**Table V** Relative risk estimates (and 95% confidence intervals) of colorectal cancer in relation to selected micronutrient intake[a], Milan, Italy, 1985–92

| *Quintile of intake* | *Colon and rectal cancer MLR*[b] | |
|---|---|---|
| *Vitamin E* | | |
| Second | 0.92 (0.74–1.15) | |
| Third | 0.97 (0.76–1.23) | |
| Fourth | 0.96 (0.74–1.26) | |
| Fifth (highest) | 1.05 (0.78–1.41) | $\chi^2_{1(trend)} = 0.0$ |
| *Folate* | | |
| Second | 1.09 (0.87–1.37) | |
| Third | 1.06 (0.82–1.36) | |
| Fourth | 1.00 (0.75–1.34) | |
| Fifth (highest) | 1.10 (0.79–1.53) | $\chi^2_{1(trend)} = 0.0$ |
| *β-Carotene* | | |
| Second | 1.00 (0.82–1.23) | |
| Third | 0.83 (0.67–1.02) | |
| Fourth | 0.71 (0.57–0.88) | |
| Fifth (highest) | 0.38 (0.30–0.50) | $\chi^2_{1(trend)} = 52.0$[c] |
| *Vitamin C* | | |
| Second | 0.68 (0.55–0.83) | |
| Third | 0.59 (0.47–0.75) | |
| Fourth | 0.58 (0.44–0.75) | |
| Fifth (highest) | 0.52 (0.38–0.70) | $\chi^2_{1(trend)} = 22.0$[c] |

[a]Reference category is the lowest quintile. [b]Multiple logistic regression estimates adjusted for age, sex, education, family history of colorectal cancer, body mass index and total energy intake plus all the above variables. [c]$P < 0.05$.

In conclusion, this analysis of a large case–control study provides support for a protective effect of a few selected nutrients on colorectal cancer risk. The protective effect persisted after allowance for several covariates, including total energy intake. Only β-carotene and ascorbic acid, however, remained significantly protective after simultaneous allowance for various micronutrients. Whether this reflects a specific (or stronger) effect of these micronutrients, rather than problems of data analysis following collinearity between micronutrients or other limitations of available data, remains open for discussion.

This work was conducted within the framework of the CNR (Italian National Research Council) Applied Projects 'Clinical Applications on Oncological Research' (Contracts No. 92.02384.PF39, No. 93.02303.PF39 and No. 92.02174.PF39), and with the contributions of the Italian Association for Cancer Research, the Italian League Against Tumours and Mrs A. Marchegiano Borgomainero. The authors wish to thank Ms Patrizia Gnagnarella for assistance with the food composition data set and Mrs Angela Simm for editorial assistance.

### References

BAKER, R.J. & NELDER, J.A. (1978). *The GLIM System*, Release 3. Numerical Algorithms Group: Oxford.

BENITO, E., SIGGELBOUT, A., BOSCH, F.X., OBRADOR, A., KALDOR, J., MULET, M. & MUÑOZ, N. (1991). Nutritional factors in colorectal cancer risk: a case–control study in Majorca. *Int. J. Cancer*, **49**, 161–167.

BRESLOW, N.E. & DAY, N.E. (1980*b*). *Statistical Methods in Cancer Research*, Vol. I, *The Analysis of Case-control Studies*. IARC Scientific Publication No. 32, IARC: Lyon.

CARNOVALE, E. & MIUCCIO, F. (1989). *Tabelle di Composizione Degli Alimenti*. Istituto Nazonale della Nutrizione: Roma.

COOPER, A.J. (1983). Biochemistry of sulfur-containing amino acids. *Annu. Rev. Biochem.*, **52**, 187–222.

FIDANZA, F. & VERDIGLIONI, N. (1988). Tabelle di composizione degli alimenti. In *Nutrizione Umana*, Fidanza, F. & Liguori, G. (eds) pp. 677–730. Idelson: Naples.

FREUDENHEIM, J.L., GRAHAM, S., MARSHALL, J.R., HAUGHY, B., CHOLEWINSKI, S. & WILKINSON, G. (1991). Folate intake and carcinogenesis of the colon and rectum. *Int. J. Epidemiol.*, **20**, 368–374.

GARLAND, C., BARRETT-CONNOR, E., ROSSOF, A.H., SHEKELLE, R.B., CRIQUI, M.H. & PAUL, O. (1985). Dietary vitamin D and calcium and risk of colorectal cancer: a 19-year prospective study in men. *Lancet*, **i**, 307–309.

GIOVANNUCCI, E., STAMPFER, M.J., COLDITZ, G.A., RIMM, E.B., TRICHOPOULOS, D., ROSNER, B.A., SPEIZER, F.E. & WILLETT, W.C. (1993). Folate, methionine, and alcohol intake and risk of colorectal adenoma. *J. Natl Cancer Inst.*, **85**, 875–884.

ISCOVICH, J.M., L'ABBÉ, K.A., CASTELLETO, R., BERNEDO, A., CHOPITA, N.A., JMELNITZSKY, A.C., KALDOR, J. & HOWE, G.R. (1992). Colon cancer in Argentina. II. Risk from fibre, fat and nutrients. *Int. J. Cancer*, **51**, 858–861.

LA VECCHIA, C., NEGRI, E., DECARLI, A., D'AVANZO, B., GALLOTTI, L., GENTILE, A. & FRANCESCHI, S. (1988). A case–control study of diet and colorectal cancer in northern Italy. *Int. J. Cancer*, **41**, 492–498.

LONGNECKER, M.P., MARTIN MORENO, J.M., KENEKT, P., NOMURA, A.M.Y., SCHOBER, S.E., STAHELIN, H.B., WALD, N.J., GEY, K.F. & WILLETT, W.C. (1992). Serum alpha-tocopherol concentration in relation to subsequent colorectal cancer: pooled data from five cohorts. *J. Natl Cancer Inst.*, **84**, 430–435.

MANTEL, N. & HAENSZEL, W. (1959). Statistical aspects of the analysis of data from retrospective studies of disease. *J. Natl Cancer Inst.*, **22**, 719–748.

NEGRI, E., LA VECCHIA, C., D'AVANZO, B. & FRANCESCHI, S. (1990). Calcium, dairy products, and colorectal cancer. *Nutr. Cancer*, **13**, 255–262.

NEWMARK, H.L. & LIPKIN, M. (1992). Calcium, vitamin D, and colon cancer. *Cancer Res.*, **52**, 2067s–2070s.

NEWMARK, H.L., WARGOVICH, M.J. & BRUCE, W.R. (1984). Colon cancer and dietary fat, phosphate, and calcium: a hypothesis. *J. Natl Cancer Inst.*, **72**, 1323–1325.

PAUL, A.A. & SOUTHGATE, D.A. (1980). *The Composition of Foods*, 4th revised edn. HMSO: London.

PETERS, R.K., PIKE, M.C., GARABRANT, D. & MACK, T.M. (1992). Diet and colon cancer in Los Angeles country, California. *Cancer Causes Control*, **3**, 457–473.

KUNE, S., KUNE, G.A. & WATSON, L.F. (1987). Case–control study of dietary etiological factors: the Melbourne colorectal cancer study. *Nutr. Cancer*, **9**, 21–42.

SORENSON, A.W., SLATTERY, M.L. & FORD, M.H. (1988). Calcium and colon cancer: a review. *Nutr. Cancer*, **11**, 135–145.

SOUCI, S.W., FACHMANN, W. & KRAUT, H. (1988). *Food Composition and Nutrition Tables, 1989/90*. Wissenschaftliche verlagsgesellschaft: Stuttgart.

WILLETT, W.C. (1989). The search for the causes of breast and colon cancer. *Nature*, **338**, 389–394.

WILLETT, W.C. & STAMPFER, M.J. (1986). Total energy intake: implications for epidemiological analyses. *Am. J. Epidemiol.*, **124**, 17–27.